# Peroxidan Plays a Tumor-Promoting Role in Oral Squamous Cell Carcinoma

**DOI:** 10.3390/ijms21155416

**Published:** 2020-07-30

**Authors:** Miyako Kurihara-Shimomura, Tomonori Sasahira, Hiroyuki Shimomura, Tadaaki Kirita

**Affiliations:** 1 Department of Oral and Maxillofacial Surgery, Nara Medical University, 840 Shijo-cho, Kashihara, Nara 634-8521, Japan; miyako@naramed-u.ac.jp (M.K.-S.); hiroz@naramed-u.ac.jp (H.S.); 2 Department of Molecular Pathology, Nara Medical University, 840 Shijo-cho, Kashihara, Nara 634-8521, Japan; tkirita@naramed-u.ac.jp

**Keywords:** oral cancer, PXDN, nodal metastasis, invasion, prognosis

## Abstract

Despite dramatic progress in cancer diagnosis and treatment, the five-year survival rate of oral squamous cell carcinoma (OSCC) is still only about 50%. Thus, the need for elucidating the molecular mechanisms underlying OSCC is urgent. We previously identified the peroxidasin gene (*PXDN*) as one of several novel genes associated with OSCC. Although the PXDN protein is known to act as a tumor-promoting factor associated with the Warburg effect, its function and role in OSCC are poorly understood. In this study, we investigated the expression, function, and relationship with the Warburg effect of PXDN in OSCC. In immunohistochemical analysis of OSCC specimens, we observed that elevated PXDN expression correlated with lymph node metastasis and a diffuse invasion pattern. High PXDN expression was confirmed as an independent predictor of poor prognosis by multivariate analysis. The PXDN expression level correlated positively with that of pyruvate kinase (PKM2) and heme oxygenase-1 (HMOX1) and with lactate and ATP production. No relationship between PXDN expression and mitochondrial activation was observed, and PXDN expression correlated inversely with reactive oxygen species (ROS) production. These results suggest that PXDN might be a tumor progression factor causing a Warburg-like effect in OSCC.

## 1. Introduction

Oral squamous cell carcinoma (OSCC) is a highly aggressive tumor, with 355,000 new cases and 177,000 deaths worldwide predicted in 2018 [1]. The main risk factors for OSCC include tobacco, alcohol, and betel quid use, human papillomavirus and candida infection, vitamin/mineral deficiencies, and immunosuppression [2]. Despite remarkable advances in the treatment of OSCC and increases in our understanding of its molecular biology, five-year survival rates over the past three decades have remained at approximately 50% [3]. Therefore, further elucidation of the molecular mechanisms underlying OSCC is needed.

We previously identified several candidate genes involved in the progression of OSCC using cDNA microarray [4]. Among the overexpressed candidate genes is the extracellular matrix (ECM) protein peroxidasin (PXDN). This protein belongs to a family of heme-containing peroxidases that catalyze the production of reactive oxygen species (ROS), including hydrogen peroxide [5,6]. Overexpression of PXDN is found in malignant glioma [7], metastatic melanoma [8], esophageal cancer [9], and bladder cancer [10]. In ovarian cancer, PXDN is associated with poor prognosis and promotes cell proliferation, invasion, and migration through activation of the phosphoinositide 3-kinase (PI3K)/Akt pathway [11]. Co-expression of PXDN and heme oxigenase-1 (HO-1) promotes the proliferation and invasion of cancer cells [12]. Thus, PXDN may possess tumor-promoting activities.

Cancer cells suppress mitochondrial activity, shift glucose metabolism to primarily glycolysis, and produce lactate and adenosine triphosphate (ATP) even in the presence of sufficient oxygen. These alterations in metabolism, known as the Warburg effect, are believed to induce the generation of ROS and promote cancer progression [13]. Under aerobic conditions, normal cells produce ATP from glucose-derived pyruvate by mitochondrial oxidative phosphorylation, while the primary source of ATP in cancer cells is aerobic glycolysis [14]. A recent report suggests that PXDN promotes prostate cancer cell tumorigenicity and viability by eliminating ROS to suppress oxidative stress and apoptosis via the Warburg effect [6]. Pyruvate kinase (PK), a glycolytic enzyme that catalyzes the last step in glycolysis, is present in mammals in four PK isoforms: PKL, PKR, PKM1, and PKM2. PKM2 is normally downregulated in response to ROS [15]. The transition from PKM1 to PKM2 expression is observed in many cancer cells [16]. The Warburg effect increases PKM2 expression and promotes lactate production under hypoxic conditions, promoting tumor growth [17].

We previously observed that in OSCC, PKM2 overexpression is associated with cell growth, invasion, and the inhibition of apoptosis via the Warburg effect [18]. PKM2 activation status may be an indicator of tumor-promoting PXDN function. However, little is known about the function of PXDN in OSCC. The purpose of this study is to investigate the expression and function of PXDN in OSCC and to clarify its relationship with the Warburg effect.

## 2. Results

### 2.1. PXDN Expression in OSCC Specimens

**Figure 1:**
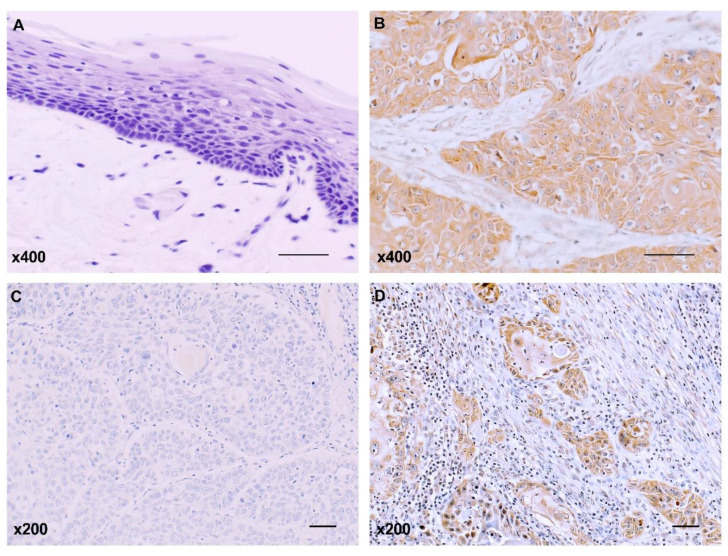
Immunostaining of peroxidasin (PXDN) in non-neoplastic oral mucosa (**A**) and oral squamous cell carcinoma (OSCC) (**B**). PXDN was more highly expressed in the diffuse invasion pattern (**D**) than in the nonaggressive invasion pattern (**C**) (*p* = 0.0307). Scale bar 100 μm.

Tissue samples from 111 OSCC patients were immunohistochemically stained for PXDN expression. Subsites of primary OSCC included the tongue (49 patients), lower gingiva (29 patients), upper gingiva (15 patients), oral floor (8 patients), buccal mucosa (6 patients), and hard palate (4 patients). T classification of these tumors was T1 (*n* = 15), T2 (*n* = 53), T3 (*n* = 27), or T4 (*n* = 16). Patient clinical stages were as follows: stage I, 15 patients; stage II, 40 patients; stage III, 35 patients; and stage IV, 21 patients. Thirty-seven patients had pathology-confirmed nodal metastasis. Immunostaining for PXDN was negative or extremely weak in the adjacent noncancerous oral mucosa (Figure 1A), while cytoplasmic expression of PXDN was detected in 42 of 111 OSCC samples (37.8%) (Figure 1B). As shown in Table 1, high PXDN expression was significantly associated with the presence of lymph node metastasis (*p* = 0.0065). PXDN immunoreactivity was observed in 28.4% of non-metastatic cases, whereas 56.8% of metastatic cancers expressed PXDN. In addition, PXDN expression in OSCC was a significant influence on the mode of invasion based on the pattern of invasion (POI) (*p* = 0.0307) [19,20,21]. PXDN expression was observed in 24.4% of patients (10/41) with a nonaggressive invasion pattern (Figure 1C) and in 45.7% of patients (32/70) with a diffuse invasion pattern (Figure 1D). No significant relationship was found between PXDN expression and other clinicopathological parameters.

### 2.2. PXDN mRNA Expression in OSCC Specimens

**Figure 2:**
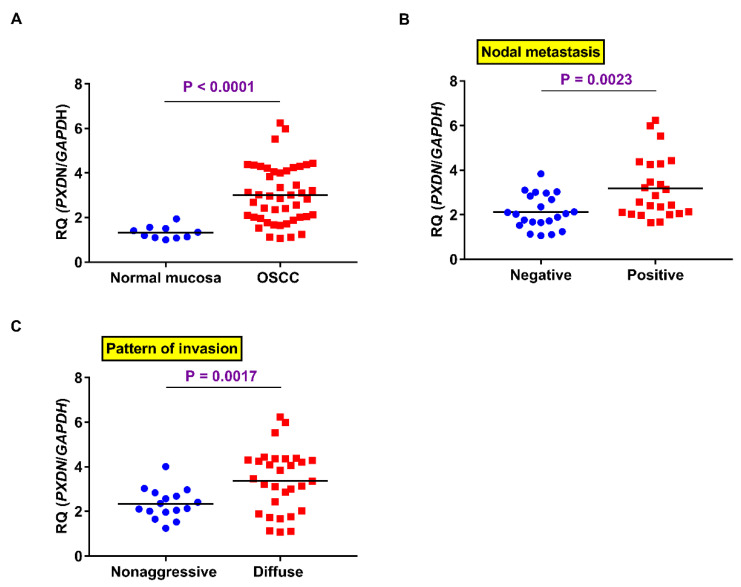
Expression of *PXDN* mRNA in OSCC samples. *PXDN* expression in OSCC was elevated as compared to normal oral mucosa (*p* < 0.0001) (**A**) and significantly positively associated with nodal metastasis (*p* = 0.0023) (**B**) and pattern of invasion (POI) (*p* = 0.0017) (**C**).

We next confirmed gene expression of *PXDN* in OSCC samples (*n* = 46). PCR analysis of *PXDN* gene expression showed that PXDN expression levels were higher in OSCC samples than in normal mucosa (*p* < 0.0001) (Figure 2A). Significantly higher PXDN expression was observed in patients with lymph node metastasis (*n* = 22) than in those without (*n* = 24) (*p* = 0.0023) (Figure 2B). Furthermore, the level of *PXDN* gene expression was higher in cases with a diffuse infiltration pattern than in those without (*n* = 30) (*p* = 0.0017) (Figure 2C). No significant relationship was observed between *PXDN* expression and other parameters.

### 2.3. Associations between PXDN Expression and Prognosis in OSCC

**Figure 3:**
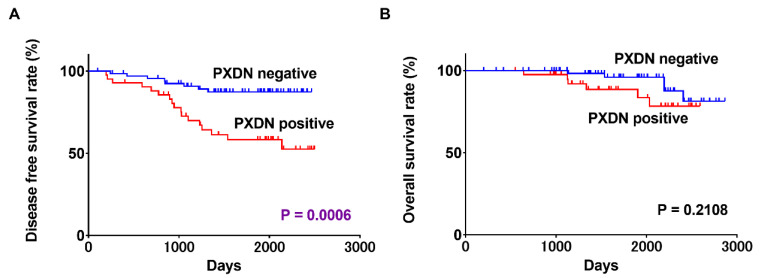
Disease-free survival (DFS) (**A**) and overall survival (OS) (**B**) of OSCC patients.

During the follow-up period, 26 of the 111 patients experienced a local or metastatic recurrence of cancer. Disease-free survival (DFS) curves constructed using the Kaplan–Meier method showed that patients with PXDN expression had a significantly shorter time to relapse than did those without (*p* = 0.0013) (Figure 3A). However, PXDN expression was not significantly associated with overall survival (OS) in patients with OSCC (Figure 3B). Table 2 shows the results of univariate and multivariate survival analysis using the Cox proportional hazards model. In univariate analysis, nodal metastasis (*p* = 0.0007), diffuse invasion pattern (*p* = 0.0307), and PXDN expression level (*p* = 0.0018) were associated with a poor outcome in OSCC patients. Of these parameters, nodal metastasis (*p* = 0.0342) and PXDN overexpression (*p* = 0.0214) remained independent predictors of worse prognosis in OSCC after multivariate analysis.

### 2.4. Relevance of Expression Levels of PXDN and HMOX1 or PKM2 in OSCC

**Figure 4:**
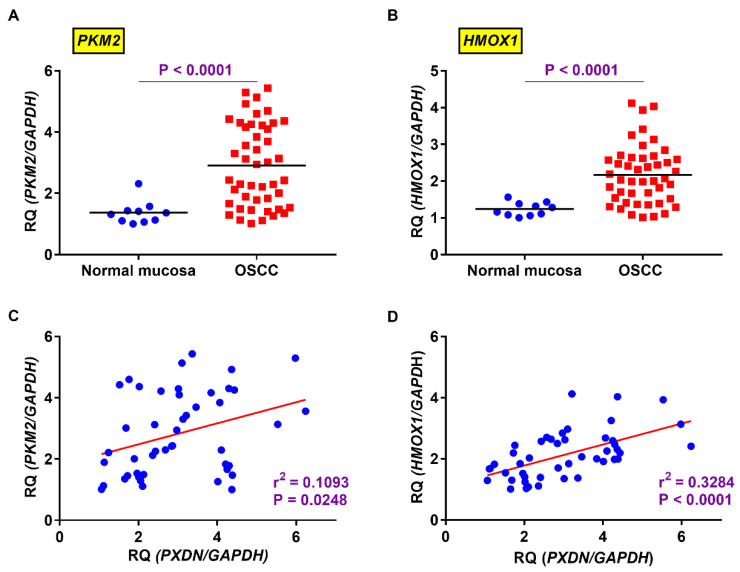
Expression levels of *heme oxigenase-1 (HMOX1)* (*p* < 0.0001) (**A**) and *pyruvate kinase M2 (PKM2)* (*p* < 0.0001) (**B**) were significantly higher in OSCC than in normal mucosa. In OSCC, the expression level of *PXDN* correlated positively with that of *PKM2* (*p* = 0.0248) (**C**) and *HMOX1*l (*p* < 0.0001) (**D**) *Glyceraldehyde-3-phosphate dehydrogenase* (*GAPDH*) mRNA was used as the internal control.

PXDN has been reported to promote cancer invasion when co-expressed with HO-1 [12]. We examined the expression levels of PXDN, HMOX1 (HO-1 gene), and PKM2 in OSCC. *HMOX*1 (*p* < 0.0001) (Figure 4A) and *PKM2* (*p* < 0.0001) (Figure 4B) were highly expressed in OSCC tissues as compared to normal mucosa. The expression level of PXDN in OSCC was significantly associated with that of *HMOX1* (*p* < 0.0001) (Figure 4C) and *PKM2* (*p* = 0.0248) (Figure 4D).

### 2.5. Comparison of PXDN Expression and Production of Lactate, ATP, and ROS in OSCC and Normal Mucosa

**Figure 5:**
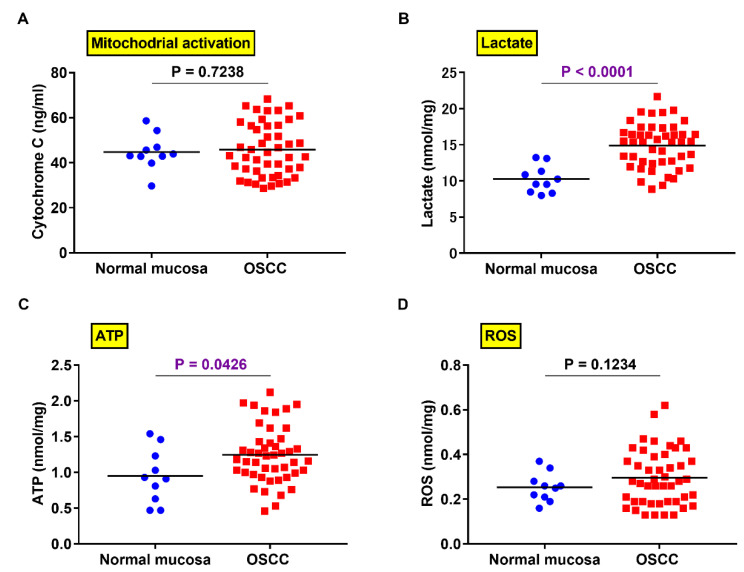
Differences in PXDN expression and lactate, ATP, and reactive oxygen species (ROS) production between OSCC and normal mucosa. No significant difference was found in the activation status of mitochondria (**A**) or reactive oxygen species (ROS) production (**D**) between OSCC and normal mucosa. The production of lactic acid (*p* < 0.0001) (**B**) and adenosine triphosphate (ATP) (*p* = 0.0426) (**C**) in OSCC was higher in OSCC than in non-neoplastic oral mucosa.

Since lactic acid and ATP are produced under aerobic conditions by the Warburg effect [13], we next measured their levels in OSCC. Although mitochondrial activation did not differ significantly between OSCC and normal mucosa (Figure 5A), the production of lactic acid (*p* < 0.0001) (Figure 5B) and ATP (*p* = 0.00426) (Figure 5C) was higher in OSCC tissues than in normal oral mucosa. No statistically significant difference was observed in ROS production between OSCC and normal mucosa (Figure 5D).

### 2.6. Relationship between PXDN Expression and Production of Lactate, ATP, and ROS in OSCC Specimens

**Figure 6:**
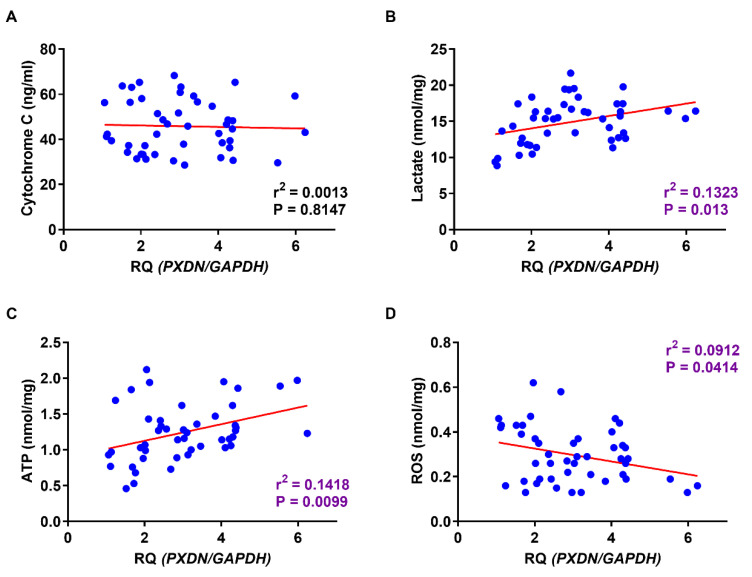
Relationship between expression of PXDN and factors related to Warburg effect in OSCC. Although a significant association between *PXDN* expression and mitochondrial activation in OSCC was not indicted (**A**), lactate (*p* = 0.013) (**B**) and ATP production (*p* = 0.0099) (**C**) correlated strongly with *PXDN* expression levels in OSCC. The expression level of *PXDN* in OSCC correlated inversely with ROS production (*p* = 0.0414) (**D**).

Finally, we analyzed the relationship between *PXDN* expression and lactate, ATP, and ROS production in OSCC. PXDN expression did not correlate with mitochondrial activation (Figure 6A) but did correlate with the production of lactate (*p* = 0.013) (Figure 6B) and ATP (*p* = 0.0099) (Figure 6C). An inverse relationship was observed between PXDN expression and ROS production (*p* = 0.0414) (Figure 6D). Together, these results suggest that PXDN might promote OSCC by affecting cancer cell metabolism.

## 3. Discussion

In the present study, we observed that PXDN expression was associated with lymph node metastasis, invasion pattern, and poor prognosis in OSCC.

The results of this study are consistent with a previous report that PXDN is involved in the Warburg-like effect [6]. The Warburg effect on cancer cells is believed to promote cancer invasion and metastasis and is one of the 10 hallmarks of cancer [13,18,22]. However, the Warburg effect that occurs in the early stage of carcinogenesis has been reported to act as a tumor suppressor [23]. Cancer cells were observed to increase the Warburg effect in cancer-associated fibroblasts by increasing oxidative stress and autophagy and using the generated lactate and pyruvate for ATP production in mitochondria (reverse Warburg effect) [24]. Whether the Warburg effect directly promotes tumor progression or is a secondary change caused by carcinogenesis is unclear.

The conversion of PKM1 to PKM2 is found in many malignancies [16]. We previously reported that PKM2 is overexpressed in OSCC compared to normal oral mucosa as a result of a Warburg-like effect and is involved in tumor progression, hypoxia induction, cell growth, and invasion and is associated with the Ki-67 labeling index [18]. In addition, the Warburg effect increases PKM2 expression and promotes lactate production [17]. The expression levels of *PXDN* and *PKM2* correlated significantly in this study. Mitochondrial activity is suppressed by the Warburg effect in cancer cells, resulting in accelerated lactate production by aerobic glycolysis [13,14]. Accordingly, we observed that elevated lactate and ATP production in OSCC correlated with *PXDN* expression. However, mitochondrial activation was not associated with *PXDN* expression. Although mitochondrial dysfunction is thought to decrease ATP production and promote cancer progression, poor-quality mitochondria accumulate in cancer cells [25]. Despite the fact that only two molecules of ATP are produced during glycolysis as compared to 36 molecules during mitochondrial oxidative phosphorylation, the rate of ATP synthesis is overwhelmingly faster in glycolysis [14]. Indeed, in an extracellular glucose-rich environment, cancer cells overproduce ATP via glycolysis-dependent metabolism [23]. Furthermore, a recent study showed that mitochondrial hypofunction is not essential for the occurrence of the Warburg effect [26]. However, distinguishing between normal and aberrant mitochondrial function is difficult, and the experiments investigating mitochondria in the present study were not successful. The detailed role of mitochondria in the action of PXDN in cancer requires further study.

The Warburg effect can cause a decrease in intracellular ROS [13,27,28]. Our present study shows that PXDN expression correlated significantly with low ROS production levels in OSCC specimens. ROS play conflicting roles in cancer, exhibiting tumor-promoting or -suppressive activities depending on the conditions [29]. Because the ROS produced by defective mitochondria promote cancer initiation and progression, decreasing oxidative stress was considered a potential means of preventing and treating cancer [30,31]. However, large multicenter clinical trials and animal studies have shown that the administration of antioxidants unexpectedly increases the incidence of cancer [32,33]. Recent reports suggest that excessive ROS accumulation in tumor cells suppresses cancer progression by inducing growth arrest and cell death; further, cancer cells with ROS levels normalized by antioxidants gave rise to distant metastasis in murine melanoma models [34,35]. Further, moderate ROS levels are reported to accelerate proliferative signaling, while higher levels induce cancer cell death [13]. PXDN may contribute to cancer progression by adjusting the production of ROS to low concentrations.

Stromal invasion by cancer cells requires the following steps: (1) loosening of tumor cell–cell adhesion, (2) degradation and destruction of the ECM, (3) action on the ECM proteins of tumor cells, (4) increased tumor cell motility and migration [22,36]. The mode of invasion in OSCC remains uncertain. As a uniform measure, studies of cancer invasion classify invasion patterns using the POI system [19]. Under this system, POI 4 and 5 designate a diffuse invasion pattern, which is associated with lymph node metastasis and poor prognosis [19,20,21]. Our results are consistent with this finding, with univariate analysis showing that patients with tumors of POI 4 and 5 had a poor prognosis.

PXDN is a cell-surface peroxidase associated with the ECM [6]. In cancer cells, interaction between PXDN and HO-1 promotes invasion by attenuating expression of the ECM proteins fibronectin and laminin [12]. Here, we observed that PXDN expression correlated with a diffuse invasion pattern, nodal metastasis, and *HMOX1* expression levels in OSCC specimens. Induction of the epithelial–mesenchymal transition (EMT) by factors such as Snail plays a pivotal role in cancer invasion and metastasis [36]. High expression of PXDN has been confirmed in prostate cancer cells overexpressing Snail [37]. However, PXDN is reported to be negatively regulated by Snail activation during the EMT in cervical cancer cells [38]. Further study is required to determine the mechanism of PXDN action during the EMT in cancer cells. Further studies are also needed to determine the role of the interaction between PXDN and HO-1, reported to have antioxidant activity [39], on the Warburg effect and ROS production.

Together, our results indicate that PXDN acts as a tumor-promoting factor in OSCC. Therefore, PXDN might be a new predictive marker for OSCC progression. However, a full understanding of the role of PXDN in OSCC and its mechanism of action requires further in vivo and in vitro study.

## 4. Materials and Methods

### 4.1. Surgical Specimens

This study was performed according to the ethical standards presented in the Declaration of Helsinki and was approved by the Medical Ethics Committee of Nara Medical University (approval number, 719, 19 August 2013). Primary OSCC specimens (*n* = 111) were collected from 111 patients (61 males, 50 females) aged 44–89 years (mean, 68.3 years) at Nara Medical University Hospital, Kashihara, Japan. The formalin-fixed and paraffin-embedded specimens from all 111 patients were analyzed immunohistochemically; in addition, samples of OSCC tissue from 46 patients (28 males and 18 females; age range, 48–81 years (mean, 65.9 years)) was frozen, and samples of nontumor oral mucosa were retrieved from 10 patients (6 males and 4 females; age range, 32–56 years (mean, 45.2 years)). The fresh-frozen samples were used for gene expression analysis and enzyme-linked immunosorbent assay (ELISA). None of the patients had undergone radiotherapy or chemotherapy before surgical resection. The tumor stage and the histological grade of OSCC were classified according to the Union for International Cancer Control (UICC) TNM system, 8th edition, and the World Health Organization criteria, respectively. The POI was classified based on previous reports, as follows: Pattern 1, pushing tumor border with smooth edges; Pattern 2, code-like, finger-shaped, or bundle of tumor growth; Pattern 3, invasive tumor nests consisting of 15 or more cells; Pattern 4, small invasive tumor nests with less than 15 cells; Pattern 5, tumor satellites >1 mm away from the main tumor [20]. The patterns were further classified as nonaggressive (POI 1–3) or diffuse invasive (POI 4–5) [19,21]. The follow-up period was 101–2499 days (mean, 1571 days; median, 1701 days).

### 4.2. Immunohistochemistry

Consecutive 3 μm sections were cut from each block, and immunohistochemical analysis was performed by using the EnVision+ Dual Link System (Dako, Carpinteria, CA, USA). Antigen retrieval was performed by microwave treatment (95 °C) in citrate buffer (pH 6.0) for 45 min. Anti-PXDN antibody (Santa Cruz Biotechnology, Santa Cruz, CA, USA) diluted to 0.5 μg/mL was used as the primary antibody. After 2 h of incubation at room temperature, the sections were incubated with a secondary antibody for 30 min. The specimens were color-developed with diaminobenzidine (DAB) solution (Dako) and counterstained with Mayer’s hematoxylin (Sigma-Aldrich, St. Louis, MO, USA).

### 4.3. Evaluation of Immunohistochemistry

Immunostaining was scored according to the percentage of immunopositive cells [15], as follows: Score 0, 0%; 1, 1–10%; 2, 11–35%; 3, 36–70%; and 4, 71–100%. Intensity scores were defined according to the immunostaining strength: 0, negative; 1, weak; 2, intermediate; and 3, strong. The total score was calculated as the sum of the proportional and intensity scores (range, 0–7). Immunoreactivity was classified into four categories according to the total score: Grade 0, total score of 0; Grade 1, total score of 2–4; Grade 2, total score of 5–6; and Grade 3, total score of 7. Tissue samples with grade 2 or 3 immunoreactivity were regarded as having high PXDN expression. Each specimen was scored in five distinct areas at 200× magnification, and the resultant five scores were averaged and rounded to the nearest whole number.

### 4.4. Quantitative Gene Expression Analysis

Total RNA was extracted using the RNeasy FFPE Kit (Qiagen, Venlo, The Netherlands), and 1 μg of total RNA was converted to cDNA with the ReverTra Ace qRT kit (Toyobo, Osaka, Japan). Quantitative reverse transcription-polymerase chain reaction (qRT-PCR) was performed using the StepOne Plus Real-Time PCR System (Applied Biosystems, Waltham, MA, USA) with the relative standard curve quantification method. TaqMan Gene Expression Assays of *PXDN, PKM2, HMOX1,* and *glyceraldehyde-3-phosphate dehydrogenase* (*GAPDH*) were purchased from Applied Biosystems. All PCR experiments were carried out in triplicate.

### 4.5. ELISA and Measurement of ATP and ROS Production

Protein was extracted using M-PER mammalian protein extraction reagent (Thermo Fisher Scientific, Rockford, IL, USA). Human Cytochrome C ELISA Kit (Abcam, Cambridge, Cambridgeshire, UK) was used to quantify the mitochondrial activity. The production of lactate, ATP, and ROS was determined using the Lactate Assay Kit-WST (Dojindo, Kumamoto, Japan), ATP Assay Kit (Abnova), and Lipid Peroxidation (MDA) Assay Kit (Biovision), respectively.

### 4.6. Statistical Analysis

Statistical analyses were performed using Fisher’s exact test, Student’s t-test, Welch’s t-test, and the Mann–Whitney U-test. The DFS and OS were analyzed using the Kaplan–Meier method and compared between groups using the log-rank test. Univariate and multivariate analyses were carried out using the Cox proportional hazards model (described as a hazard ratio with 95% confidence intervals (95% CI), together with the *p* value). All of the tests were two-sided, and *p* < 0.05 was considered statistically significant.

## Figures and Tables

**Table 1:** Relationship between PXDN expression and clinicopathological parameters.

Parameters		PXDN Expression		*p* Value *
		Negative (%)	Positive (%)	
Gender	Male	40 (65.6)	21 (34.4)	
	Female	29 (58)	21 (42)	0.4376
Age	<65	26 (65)	14 (35)	
	>65	43 (60.6)	28 (39.4)	0.6877
Site	Tongue	29(59.2)	20 (40.8)	
	Other	40 (64.5)	22 (35.3)	0.6938
Histological differentiation	Well	34 (58.6)	24 (41.4)	
	Moderately, Poorly	35 (66)	18 (34)	0.4405
T classification	T1–T2	40 (58.8)	28 (41.2)	
	T3–T4	29 (67.4)	14 (32.6)	0.4242
Clinical stage	I–II	40 (58.8)	28 (41.2)	
	III–IV	29 (67.4)	14 (32.6)	0.4242
Nodal metastasis	Negative	53 (71.6)	21 (28.4)	
	Positive	16 (43.2)	21 (56.8)	0.0065
POI	Nonaggressive pattern	31 (75.6)	10 (24.4)	
	Diffuse invasion pattern	38 (54.3)	32 (45.7)	0.0277

Relationship between expression of PXDN and each factor was calculated by Fisher’s exact test. T classification and clinical stage were classified according to the tumor, node, metastasis (TNM) classification. Pattern of invasion (POI) was divided into nonaggressive pattern (POI 1–3) and diffuse invasion pattern (POI 4-5). * *p* value < 0.05 was regarded as statistically significant.

**Table 2:** Univariate and multivariate analysis of disease-free survival.

Parameters		Univariate Analysis			Multivariate Analysis		
		HR	95% CI	*p* Value	HR	95% CI	*p* Value
Gender	M	1.00					
	F	1.1734	0.5367–2.5634	0.6851			
Age	<65	1.00					
	>65	1.1734	0.8296–3.9450	0.1341			
Site	Tongue 29 (59.2)	1.00					
	Other	1.0041	0.4589–2.2311	0.9917			
Histology	Well	1.00					
	Mod, Por	1.8282	0.8324–4.2970	0.1345			
T factor	T1–T2	1.00					
	T3–T4	1.1916	0.5330–2.5817	0.6608			
Clinical stage	I–II	1.00					
	III–IV	1.8837	0.8571–4.4289	0.1163			
Nodal Metastasis	Negative	1.00			1.00		
	Positive	3.8307	1.7584–8.7592	0.0007	2.5907	1.0710–6.7677	0.0342
POI	Nonaggressive pattern	1.00			1.00		
	Diffuse invasion pattern	2.6793	1.0898–8.0350	0.030	1.4048	0.4892–4.6331	0.5393
PXDN expression	Negative	1.00			1.00		
	Positive	3.4853	1.5873–8.1899	0.0018	2.5971	1.1499–6.2578	0.0214

Univariate and multivariate analysis was calculated by means of Cox proportional hazard model. HR and 95% CI mean hazard ratio and 95% confidence intervals, respectively.

## Abbreviations

DFS	Disease-free survival
ELISA	Enzyme-linked immunosorbent assay
HO-1	Heme oxigenase-1
OSCC	Oral squamous cell carcinoma
PK	Pyruvate kinase
POI	Pattern of invasion
PXDN	Peroxidasin
ROS	Reactive oxygen species
